# Uniform excitations in magnetic nanoparticles

**DOI:** 10.3762/bjnano.1.6

**Published:** 2010-11-22

**Authors:** Steen Mørup, Cathrine Frandsen, Mikkel Fougt Hansen

**Affiliations:** 1 Department of Physics, Building 307, Technical University of Denmark, DK-2800 Kongens Lyngby, Denmark; 2 Department of Micro- and Nanotechnology, DTU Nanotech, Building 345 East, Technical University of Denmark, DK-2800 Kongens Lyngby, Denmark

**Keywords:** collective magnetic excitations, Mössbauer spectroscopy, neutron scattering, spin waves, superparamagnetic relaxation

## Abstract

We present a short review of the magnetic excitations in nanoparticles below the superparamagnetic blocking temperature. In this temperature regime, the magnetic dynamics in nanoparticles is dominated by uniform excitations, and this leads to a linear temperature dependence of the magnetization and the magnetic hyperfine field, in contrast to the Bloch *T*^3/2^ law in bulk materials. The temperature dependence of the average magnetization is conveniently studied by Mössbauer spectroscopy. The energy of the uniform excitations of magnetic nanoparticles can be studied by inelastic neutron scattering.

## Review

### Introduction

One of the most important differences between magnetic nanoparticles and the corresponding bulk materials is that the magnetic dynamics differ substantially. The magnetic anisotropy energy of a particle is proportional to the volume. For very small particles at finite temperatures it may therefore be comparable to the thermal energy. This results in superparamagnetic relaxation, i.e., thermally induced reversals of the magnetization direction. For a particle with a uniaxial anisotropy energy *E*(θ) given by the simple expression in Equation 1, the superparamagnetic relaxation time τ is given by Equation 2 [1–2].

[1]
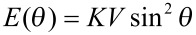


[2]
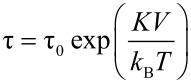


Here *K* is the magnetic anisotropy constant, *V* is the particle volume, θ is the angle between an easy axis and the (sublattice) magnetization vector, *k*_B_ is Boltzmann’s constant and *T* is the temperature. The value of τ_0_ is in the range 10^−13^–10^−9^ s. When the superparamagnetic relaxation time is long compared to the timescale of the experimental technique, the instantaneous magnetization is measured, but in the case of fast relaxation, the average value of the magnetization is measured. The superparamagnetic blocking temperature (*T*_B_) is defined as the temperature at which the superparamagnetic relaxation time equals the timescale of the experimental technique used for the study of the magnetic properties.

**Figure 1:**
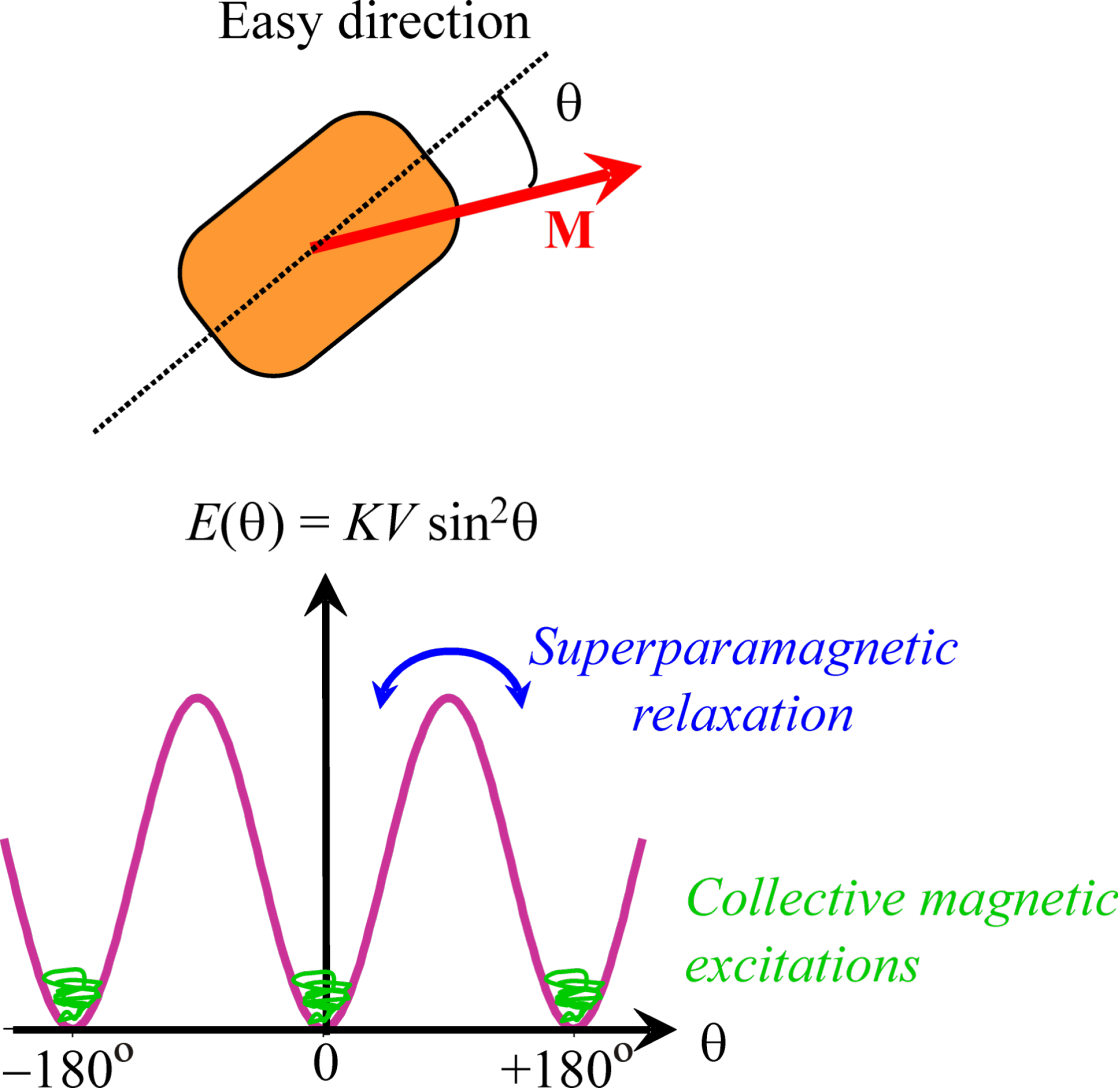
Schematic illustration of magnetic fluctuations in a nanoparticle. At low temperatures the direction of the magnetization vector **M** fluctuates near one of the easy directions (collective magnetic excitations). At higher temperatures the thermal energy can be comparable to the height, *KV*, of the energy barrier separating the easy directions, and the magnetization can fluctuate between the easy directions (superparamagnetic relaxation).

Below *T*_B_, superparamagnetic relaxation can be considered negligible, but the magnetization direction may still fluctuate in directions close to the easy axes at θ *=* 0° and θ *=* 180°*.* These fluctuations have been termed “collective magnetic excitations” [3–5]. The magnetic excitations in a nanoparticle are illustrated schematically in Figure 1.

The magnetic dynamics well below the Curie or Néel temperature in both bulk materials and nanoparticles can be described by excitation of spin waves, but the spin wave spectrum of small particles is size-dependent and this can have a substantial influence on the temperature dependence of the magnetization in nanoparticles. In this paper we give a short review of the spin dynamics in non-interacting nanoparticles below the blocking temperature.

### Ferromagnetic and ferrimagnetic nanoparticles

First, we consider a ferromagnetic or ferrimagnetic material with cubic crystal structure and lattice constant *a*_0_. The dispersion relation for spin waves, i.e., the spin wave energy 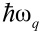 as a function of the value of the wave vector *q*, can for *a*_0_*q* << 1 be written as [6–7]

[3]
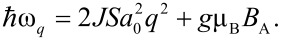


Here ω*_q_* is the angular frequency of a spin wave with wave vector *q*, *J* is the exchange constant, *S* is the atomic spin, *g* is the Landé factor, μ_B_ is the Bohr magneton, and *B*_A_ = 2*K*/*M*_0_ is the anisotropy field, where *M*_0_ is the saturation magnetization. If surface effects are negligible, the allowed values of the wave vector in a cubic nanoparticle with side length *d* are given by [7–10]

[4]
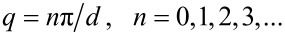


In a macroscopic crystal, the energy difference between adjacent spin wave states is small and the quantized states are well approximated by a continuous distribution of energies. Furthermore, the magnetic anisotropy is usually neglected in the calculations [9–10]. In ferromagnetic and ferrimagnetic materials at low temperatures, spin wave excitations result in a temperature dependence of the magnetization given by [10–11]

[5]
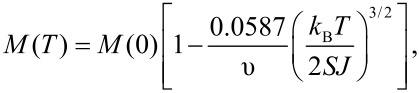


where υ is an integer, which equals 1, 2, or 4 for simple cubic, bcc or fcc lattices, respectively. Equation 5 is known as the Bloch *T*^3/2^ law.

In a nanoparticle, the value of *d* is small, and according to Equation 3 and Equation 4 this results in large energy gaps between spin wave modes with different *q*-values. The energy gap between the *n* = 0 and *n* = 1 modes is given by [7]

[6]
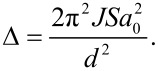


This energy gap can be on the order of 10 K or larger.

**Figure 2:**
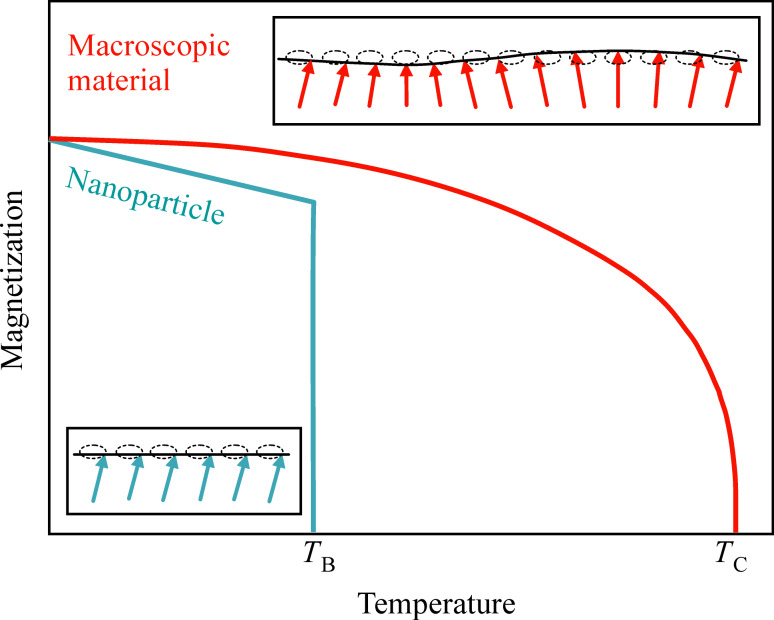
Schematic illustration of spin waves in macroscopic crystals (red arrows) and uniform excitations in nanoparticles (blue arrows) and the corresponding temperature dependence of the magnetization. *T*_B_ is the superparamagnetic blocking temperature and *T*_C_ is the Curie temperature.

In the spin wave modes with *n >* 0, the atomic spins precess such that adjacent spins form a small angle with each other. However, in the uniform (*n* = 0) spin-wave mode the atomic spins precess in unison, as illustrated in Figure 2. Due to the energy gap, given by Equation 6, the uniform mode is predominant in nanoparticles at low temperatures [8]. In the uniform mode, *q* = 0, and the energy of a particle in an excited uniform precession state is governed by the magnetic anisotropy and is given by

[7]
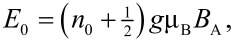


where *n*_0_ can assume the values 0, 1, 2, …

At low temperatures, the average number 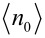 is given by [8]

[8]
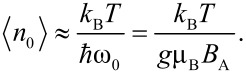


The anisotropy field may be on the order of 0.1 T or smaller. The *z*-components of the magnetic moments of neighboring precession states of the uniform mode with slightly different precession angles differ by *g*μ_B_, and the *z*-component of the magnetization at the temperature *T* is given by

[9]
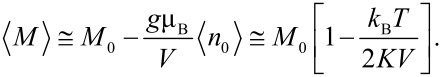


According to Equation 9, the temperature dependence of the magnetization in nanoparticles at low temperatures is independent of the exchange interaction, in contrast to bulk materials (Equation 5). Furthermore, the magnetization depends linearly on temperature, in contrast to the Bloch *T*^3/2^ law for bulk materials, as illustrated schematically in Figure 2.

The magnetic dynamics in nanoparticles at low temperatures can be described in terms of excitations of the uniform mode in combination with transitions between excited states with different values of *n*_0_, i.e., with different precession angles. These dynamics have also been termed “collective magnetic excitations” [4–5]. The contribution from the uniform mode to the temperature dependence of the magnetization of bulk materials is negligible, because of the dependence of the magnetization on the volume in Equation 9.

The temperature dependence of the magnetization of a nanoparticle can also be derived by considering the particle as a macrospin, which can be treated as a classical magnetic moment, i.e., it is assumed that the magnetization vector can point in any direction [3–5]. Below *T*_B_, the magnetization direction remains near one of the minima and the temperature dependence of the magnetization can be calculated by use of Boltzmann statistics:

[10]
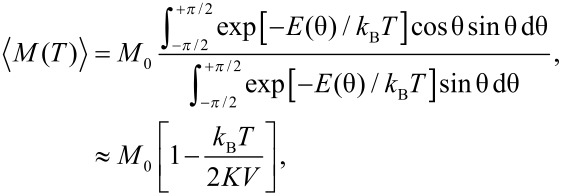


where *E*(θ) is given by Equation 1, and the latter approximation is valid at low temperatures. The linear temperature dependence of the magnetization in nanoparticles was first observed by Mössbauer spectroscopy studies of magnetite (Fe_3_O_4_) nanoparticles [3], but it has later been studied in nanoparticles of several other magnetic materials.

In Mössbauer spectroscopy studies, the magnetic hyperfine field is measured, which is proportional to the magnetization. If the magnetic fluctuations near an energy minimum are fast compared to the timescale of the technique, which is on the order of a few nanoseconds for Mössbauer spectroscopy, then the average magnetic hyperfine field is measured. The relaxation times for transitions between states with different values of *n*_0_ are much shorter than the pre-exponential factor τ_0_ in Equation 2 [12–13], which is on the order of 10^−13^–10^−9^ s. Thus, it is a good approximation to assume that the relaxation is fast compared to the timescale of Mössbauer spectroscopy, and the observed magnetic hyperfine field is then given by

**Figure 3:**
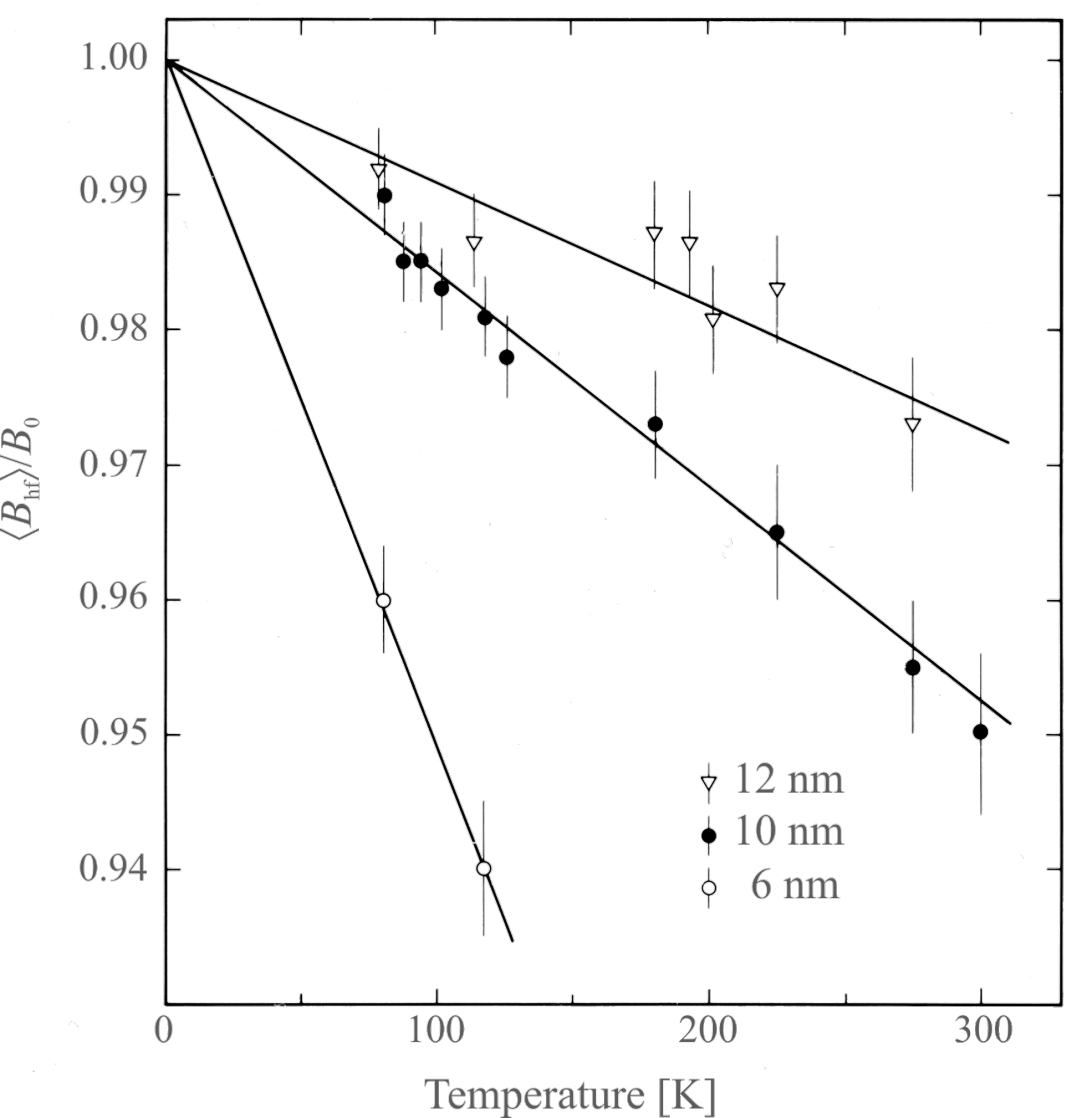
The reduced average magnetic hyperfine field 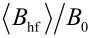 as a function of temperature for particles of magnetite with sizes of 6 nm, 10 nm and 12 nm. The solid lines are the best linear fits to the experimental data in accordance with Equation 11. [Reprinted with permission from Mørup, S.; Topsøe, H. Mössbauer Studies of Thermal Excitations in Magnetically Ordered Microcrystals, *Appl. Phys. ***1976,** *11,* 63-66. Copyright (1976) by Springer Science + Business Media.]

[11]
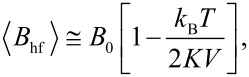


where *B*_0_ is the saturation hyperfine field. Figure 3 shows the temperature dependence of the magnetic hyperfine field of three samples of magnetite (Fe_3_O_4_) nanoparticles with different average sizes [3]. From the slopes of the fitted straight lines one can estimate the values of the magnetic anisotropy constants *K*. It was found that *K* increases with decreasing particle size. This is in accordance with the expected increase of the surface contribution to the total magnetic anisotropy [14]. A similar size dependence of the magnetic anisotropy constant has been found by Mössbauer studies in other nanoparticles, for example, maghemite (γ-Fe_2_O_3_) [15], hematite (α-Fe_2_O_3_) [16] and metallic iron (α-Fe) [17].

If a sufficiently large magnetic field *B* is applied, such that *B* >> *B*_A_, the anisotropy field in Equation 7 and Equation 8 should be replaced by the applied field. This leads to a temperature and field dependence of the magnetization given by [18]

[12]
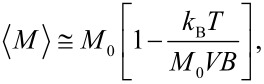


which also can be derived as the high-field approximation to the Langevin function [18]. The temperature and field dependence of the magnetic hyperfine field is given by an equivalent expression.

The timescale of inelastic neutron scattering is on the order of picoseconds, i.e., much shorter than that of Mössbauer spectroscopy, and inelastic neutron scattering can be used to measure the energy difference between adjacent uniform precession states in nanoparticles [19–22]. In inelastic neutron studies of magnetic dynamics of ferrimagnetic particles one can measure the energy distribution of neutrons that are diffracted at a scattering angle corresponding to a magnetic diffraction peak. This energy distribution is usually dominated by a large peak at zero energy, due to elastically scattered neutrons. The energy difference between neighboring precession states in the uniform mode results in satellite peaks in the energy distribution at energies ±ε_0_. These peaks are associated with transitions of the type *n*_0_ → *n*_0_ ± 1. The energy ε_0_ is given by [20]

[13]
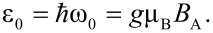


Anisotropy fields on the order of 0.1 T correspond to ε_0_ ≈ 0.01 meV. Using a neutron spectrometer with an energy resolution on the order of 0.1 meV, the satellite peaks are therefore difficult to observe, but they may be visible if a large magnetic field *B* is applied, because they are then shifted to higher energies [20]. For *B* >> *B*_A_, the energy is approximately given by an expression similar to Equation 13 with *B*_A_ replaced by *B*_._ The relative intensity of the satellite peaks is for *M*_0_*VB* >> *k*_B_*T* given by [20]

[14]
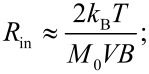


i.e., it decreases with increasing field because the uniform excitations are suppressed by the applied magnetic field. The temperature dependence of *R*_in_ is in accordance with the expected increase of the population of the uniform precession mode with increasing temperature.

**Figure 4:**
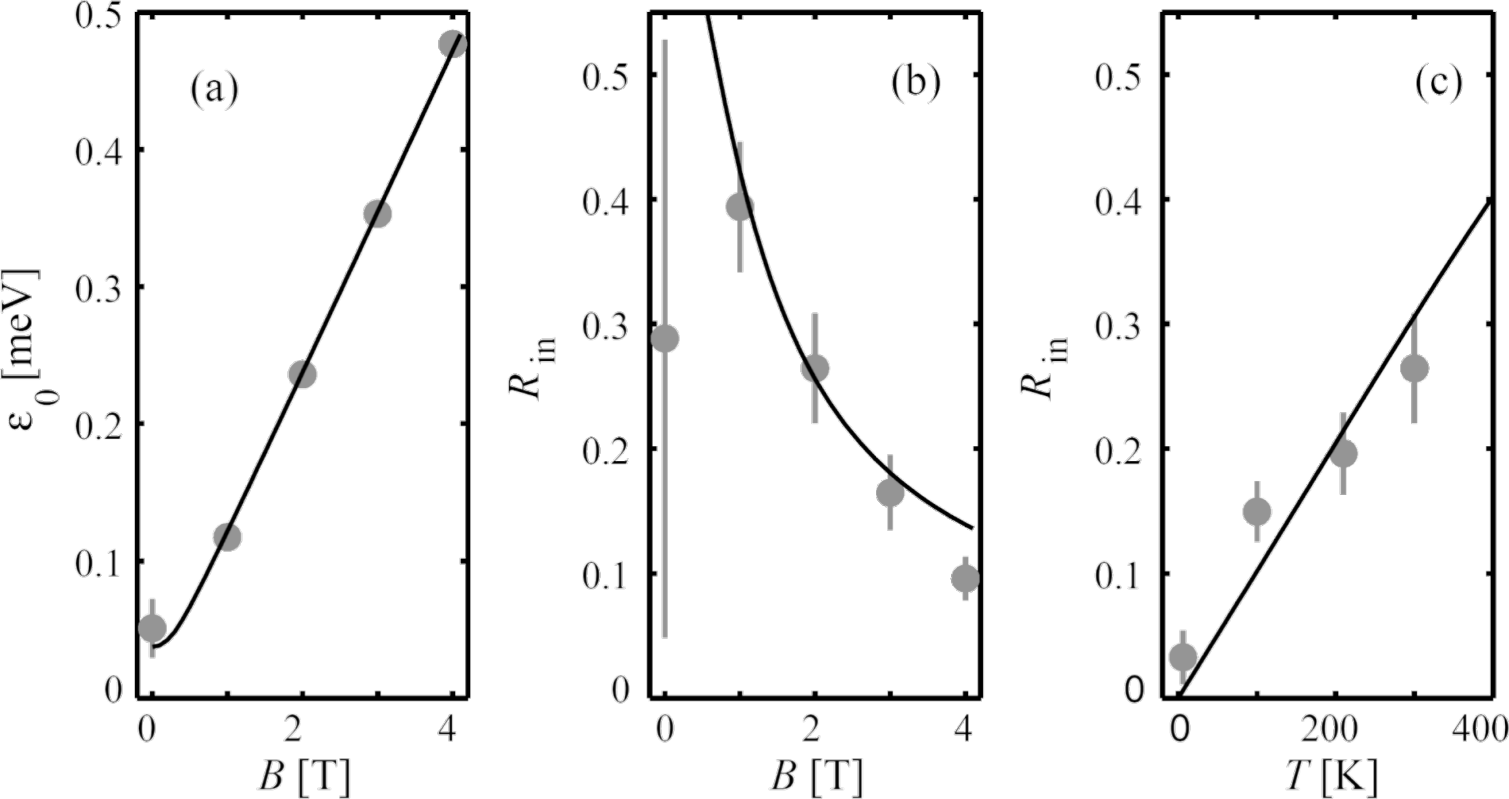
Parameters derived from inelastic neutron scattering data for 4.0 nm particles of maghemite: (a) Energy of the satellite peaks as a function of the applied magnetic field at 300 K; (b) the relative area of the inelastic peaks as a function of the applied field at 300 K; (c) the relative area of the inelastic peaks as a function of temperature at *B* = 2 T. [Reprinted with permission from Lefmann, K.; Bødker, F.; Klausen, S. N.; Hansen, M. F.; Clausen, K. N.; Lindgård, P.-A.; Mørup, S. A neutron scattering study of spin precession in ferrimagnetic maghemite nanoparticles *Europhys. Lett. ***2001,** *54*, 526–532. Copyright (2001) by EDP Sciences.]

In Figure 4, data obtained from inelastic neutron scattering studies of 4.0 nm maghemite particles [20] is shown. Figure 4a demonstrates that the energy of the satellite peaks varies almost linearly with the magnitude of the applied magnetic field, indicating that the anisotropy field is almost negligible for *B* > 1 T. By a detailed analysis of the data, *B*_A_ was estimated to be on the order of 0.3 T. Panels (b) and (c) in Figure 4 show the relative area, *R*_in_, of the satellite peaks as a function of the applied field at 300 K and as a function of temperature at *B* = 2 T, respectively, and the lines are fits by using Equation 14.

### Antiferromagnetic nanoparticles

The magnetic dynamics of nanoparticles of antiferromagnetic particles differs in several ways from that of ferro- and ferrimagnetic nanoparticles [23]. In an antiferromagnetic material with uniaxial anisotropy, the dispersion relation for spin waves is given by [10]

[15]
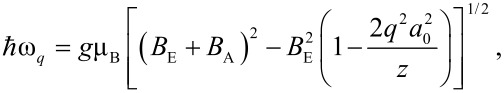


where *B*_A_ = *K/M*_s_ is the anisotropy field for an antiferromagnetic material with sublattice magnetization *M*_s_, *B*_E_ is the exchange field and *z* is the number of nearest neighbor atoms. The exchange fields of antiferromagnetic materials may be larger than 100 T, i.e., much larger than the anisotropy field. Therefore, in antiferromagnetic nanoparticles, the energy gap between the uniform mode at *n* = 0 and the *n* =1 mode is much larger than in ferro- or ferrimagnetic materials. However, in spite of the differences in the excitation energies of ferro- or ferrimagnetic particles and antiferromagnetic particles, the temperature dependence of the sublattice magnetization and the magnetic hyperfine field in antiferromagnetic nanoparticles are still given by Equation 10 and Equation 11 [8].

**Figure 5:**
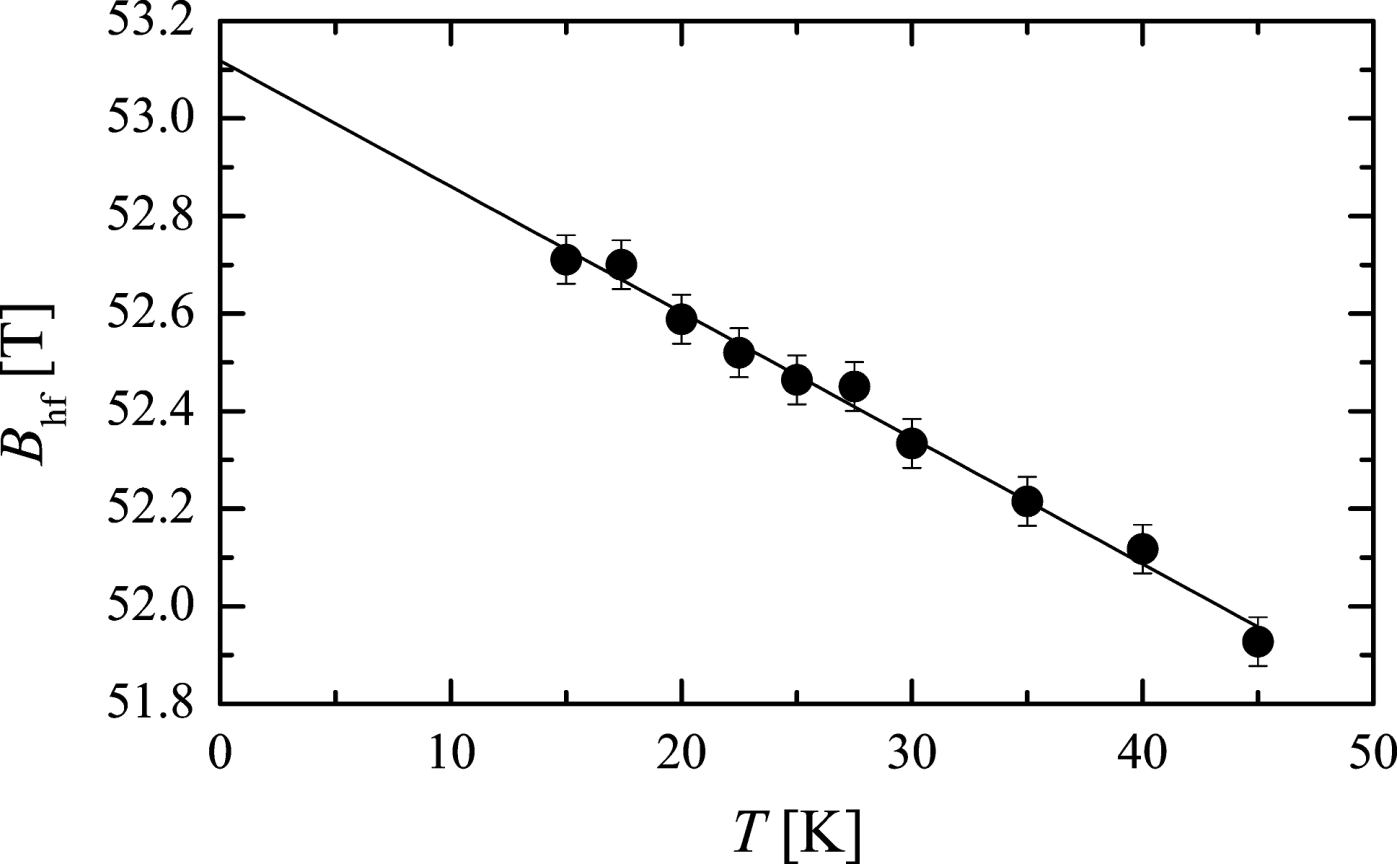
The observed median hyperfine field for 20 nm hematite nanoparticles as a function of temperature. The line is a fit in accordance with Equation 11. [Adapted from Hansen, M. F.; Bender Koch, C.; Mørup, S. The magnetic dynamics of weakly and strongly interacting hematite nanoparticles, *Phys. Rev. B ***2000,** *62,* 1124–1135. Copyright (2000) by the American Physical Society.]

As an example, Figure 5 shows the temperature dependence of the magnetic hyperfine field of 20 nm hematite nanoparticles [24]. From the slope of the linear fit of the data for the non-interacting particles, the value of the magnetic anisotropy constant can be estimated with Equation 11.

The energy difference between neighboring precession states in the uniform (*q* = 0) mode is given by [6,10,19,25]

[16]
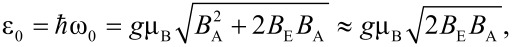


where the last approximation is valid for *B*_E_ >> *B*_A_. Because the exchange fields of typical antiferromagnetic materials are much larger than the anisotropy fields, the energy, ε_0_, can be much larger than in ferro- and ferrimagnetic nanoparticles and can more easily be resolved in inelastic neutron scattering experiments in zero applied field [19,21–22].

**Figure 6:**
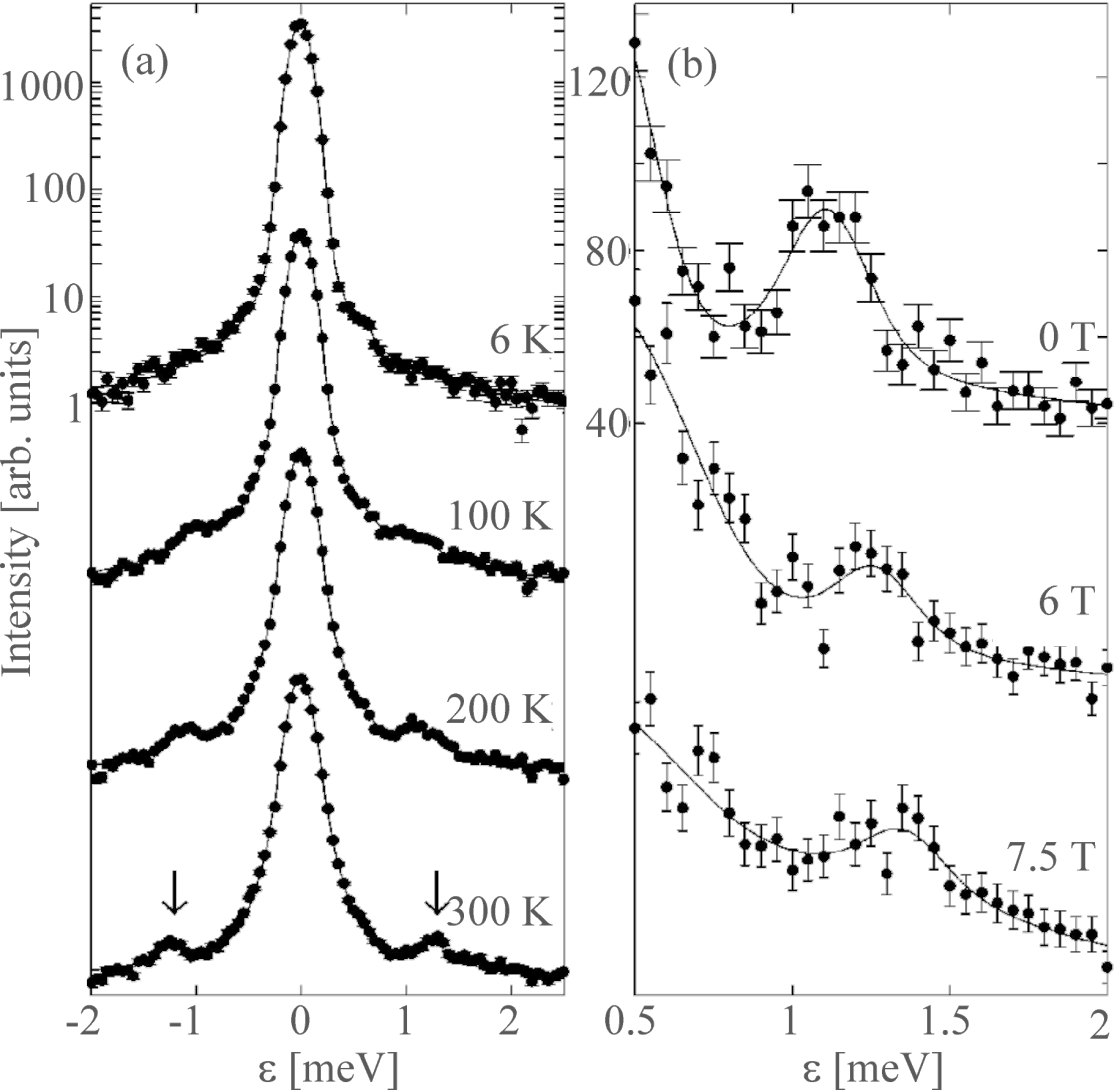
Inelastic neutron scattering data for 15 nm hematite particles measured at the scattering vector *Q* = 1.50 Å^−1^: (a) Data obtained in zero applied magnetic field at the indicated temperatures; (b) data obtained at 200 K with the indicated applied magnetic fields. [Reprinted from Klausen, S. N.; Lefmann, K.; Lindgård, P.-A.; Kuhn, L. T.; Frandsen, C.; Mørup, S.; Roessli, B.; Cavadini, N. Quantized spin waves and magnetic anisotropy in hematite nanoparticles. *Phys. Rev. B ***2004**, *70*, 214411. Copyright (2004) by the American Physical Society.]

Figure 6 shows inelastic neutron scattering data from a sample of 15 nm α-Fe_2_O_3_ nanoparticles. The data were obtained from neutrons, scattered at the scattering vector with *Q* = 1.50 Å^−1^, corresponding to the purely magnetic hexagonal (101) peak [21]. Data obtained in zero applied field as a function of temperature are shown in Figure 6a, whereas Figure 6b shows data obtained in different applied magnetic fields at 200 K. In Figure 6a, inelastic satellite peaks at energies ±ε_0_ ≈ ±1.1 meV are seen on both sides of the intense quasielastic peak. As in the data for ferrimagnetic maghemite (Figure 4) the relative area of the inelastic peaks increases with increasing temperature. At low temperatures the relative area of the inelastic peaks in zero applied field is given by [23]

[17]
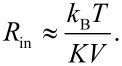


When magnetic fields are applied at 200 K, the inelastic peaks are shifted to higher energies, and their relative intensity decreases as for ferrimagnetic maghemite nanoparticles (Figure 4).

The energy of the uniform excitations in antiferromagnetic materials, Equation 16, was derived assuming that the antiferromagnetic material had zero net magnetization, but nanoparticles of antiferromagnetic materials usually have a magnetic moment because of uncompensated spins, for example, in the surface [23,26]. This can have a large influence on the excitation energy [25,27]. For example, an uncompensated magnetic moment of only around 1% of the sublattice magnetic moment can result in a decrease of the excitation energy by a factor of two [27]. Neutron studies of hematite nanoparticles [28] have shown that the effect is significant in 8 nm hematite particles, which have relatively large uncompensated moments.

**Figure 7:**
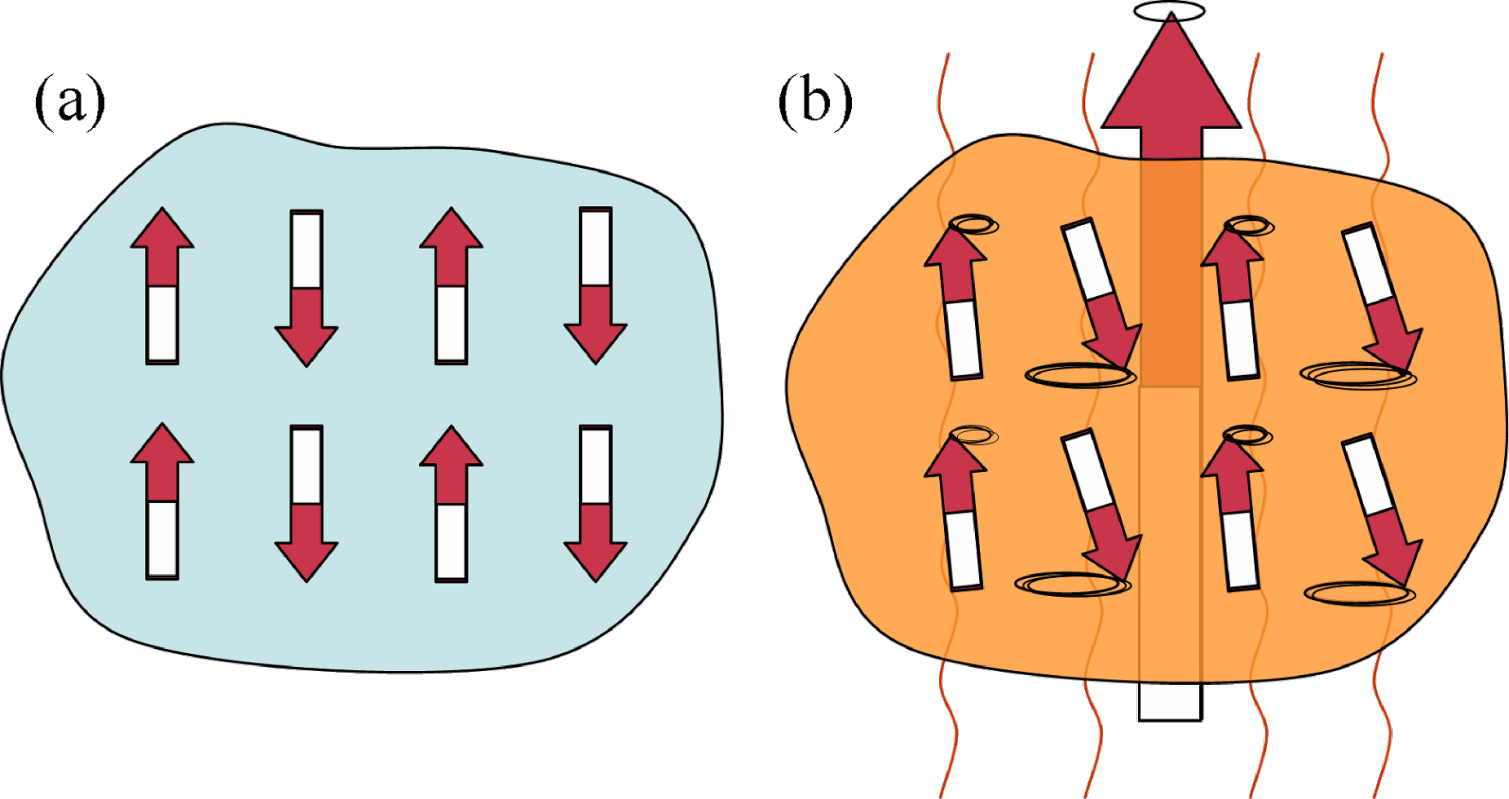
Schematic illustration of the uniform mode in antiferromagnetic nanoparticles: (a) At low temperatures the spins in the two sublattices are essentially antiparallel; (b) at higher temperatures the two sublattices are not antiparallel, but precess around an easy axis with different precession angles. This leads to a non-zero magnetic moment of the nanoparticle.

In antiferromagnetic materials, excitation of the uniform mode has interesting consequences. The spins of the two sublattices precess around the easy axis in such a way that they are not strictly antiparallel, but form different angles, θ_A_ and θ_B_, with respect to the easy axis. This is illustrated in Figure 7. For *B*_A_ << *B*_E_ the two angles are related by [29]

[18]
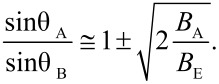


Therefore, the magnetic moments of the two sublattices do not cancel, and the nanoparticle has a net magnetic moment, which increases with increasing temperature. The contribution to the initial susceptibility from this thermoinduced magnetization is given by [30]

[19]
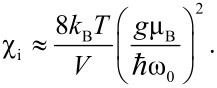


Several studies of the magnetization of antiferromagnetic nanoparticles have demonstrated an apparent increase of the magnetization with increasing temperature, which is in accordance with the model for thermoinduced magnetization [30]. However, magnetization curves of samples of antiferromagnetic nanoparticles can be significantly influenced by the distribution of magnetic moments due to uncompensated spins [31] and by the magnetic anisotropy [32], and these effects may be difficult to distinguish from the contribution from the thermoinduced magnetization.

## Conclusion

After the discovery of superparamagnetism much of the research in the field of magnetic nanoparticles has focused on superparamagnetic relaxation while the magnetic dynamics below *T*_B_ has attracted less attention. However, the quantization of the spin-wave spectrum, especially the large energy gap between the lowest (*q* = 0) excitation state and the states with *q* > 0, results in a predominance of the uniform mode in nanoparticles. This results in a linear temperature dependence of the magnetization and the magnetic hyperfine field, in contrast to the Bloch *T*^3/2^ law in bulk materials. Mössbauer spectroscopy is useful for studies of the temperature dependence of the magnetization of nanoparticles, whereas inelastic neutron scattering studies can give information on the energy of the uniform excitations.
